# Effects of Social Networking Service (SNS) Addiction on Mental Health Status in Chinese University Students: Structural Equation Modeling Approach Using a Cross-sectional Online Survey

**DOI:** 10.2196/26733

**Published:** 2021-12-08

**Authors:** Tingxuan Wang, Janet Y H Wong, Man Ping Wang, Amanda Chiu Yin Li, Sang Suk Kim, Jung Jae Lee

**Affiliations:** 1 School of Nursing The University of Hong Kong Hong Kong China; 2 Department of Medicine The University of Hong Kong Hong Kong China; 3 Red Cross College of Nursing Chung-Ang University Seoul Republic of Korea

**Keywords:** social networking service, SNS, addiction, depression, anxiety, psychosocial status, youth, mental health

## Abstract

**Background:**

Although social networking services (SNSs) have become popular among young people, problematic SNS use has also increased. However, little is known about SNS addiction and its association with SNS use patterns and mental health status.

**Objective:**

This study aims to test the mediating role of SNS addiction between SNS use patterns and mental health status among Chinese university students in Hong Kong (HK).

**Methods:**

An online cross-sectional survey was conducted using a convenience sampling method. In total, 533 university students (323 [66.9%] female, mean age [SD]=20.87 [2.68] years) were recruited from February to March 2019. Multiple linear regression was used to assess the association between SNS use and SNS addiction. Structural equation modeling (SEM) was performed to examine the pathways and associations among SNS use, SNS addiction, psychosocial status, and mental health status (including anxiety and depressive symptoms).

**Results:**

A longer time spent on SNSs per day (>3 h), a longer time spent on each SNS access (≥31 min), a higher frequency of SNS access (≤every 30 min), a longer duration of SNS use before sleeping (≥61 min), and a shorter duration from waking to first SNS use (≤5 min) were significantly associated with a higher level of SNS addiction (adjusted beta [aβ]=6.03, 95% CI 4.66-7.40; aβ=4.99, 95% CI 3.14-6.83; aβ=5.89, 95% CI 4.14-7.64; aβ=5.92, 95% CI 4.19-7.65; and aβ=3.27, 95% CI 1.73-4.82, respectively). SEM showed a significant mediating effect of SNS addiction in the relationship between SNS use and psychosocial status, and mental health status, including an indirect effect (β=0.63, 95% CI 0.37-0.93) and the total effect (β=0.44, 95% CI 0.19-0.72), while the direct effect was insignificant (β=–0.19, 95% CI –0.49 to 0.08).

**Conclusions:**

SNS use patterns were associated with SNS addiction, and SNS addiction mediated the associations between SNS use, psychosocial status, and mental health status of Chinese university students in HK. The findings suggest that screening for and addressing excessive SNS use are needed to prevent SNS addiction and mental distress among young people.

## Introduction

More than 1 billion people worldwide regularly use social networking services (SNSs), such as Facebook, Twitter, and Instagram, which are virtual communities where users interact and build online and real-life relationships [1-3]. Similar to the global trend, the number of SNS users has substantially increased from 68% in 2014 to 83% in 2018 in Hong Kong (HK) [4] which has 1 of the highest penetration rates of the internet and e-devices [5,6]. Compared with other age groups, younger age groups more popularly use SNSs, and 95% of young people in HK, aged from 10 to 24 years, used SNSs for an average of 17.7 h/week (the longest SNS use than other age groups) in 2018 [4]. As SNS use has gained popularity, problematic SNS use, such as excessive SNS use, which is a relatively long duration or frequent use of SNSs [7], has also increased among young people [8,9]. Evidence has shown that excessive SNS use has a negative impact on young people’s physical health (eg, headache, postural pain, and eye strain) [7,10], real-life social relationships [11], and academic performance [12,13]. Moreover, although SNS platforms provide more pervasive environments for individuals to share their feelings and follow other interesting friends, previous studies have reported that excessive SNS use shows a strong association with psychological distress, including depression and anxiety [13-16], such as feeling frustrated or isolated from their real life [17] and feeling vulnerable to the perceptions of others [18].

Excessive SNS use has shown significant association with addictive behaviors [19,20], including feeling constantly anxious or uneasy about one’s SNS accounts and having strong urges to use SNSs. SNS addiction is thus defined as the excessive use of SNSs that affects one’s daily activities, interpersonal relationships, and mental health [21]. Although SNS addiction has not been specifically identified by the *Diagnostic and Statistical Manual 5th Edition* (DSM-5) or the *International Classification of Diseases 11th Revision* (ICD-11), SNS addiction can be considered as 1 form of behavior addiction, reflecting excessive and compulsive symptoms [22,23], similar to internet gaming disorder. However, SNS addiction can be differentiated from other behavior addictions as users focus on interpersonal networking through SNSs. According to gratification theory, users who gain enjoyment and happiness through SNS platforms would continue to spend even more time using SNSs in pursuit of more positive gratification [24]. Therefore, SNS addiction may result from gratification attained through online surfing and through virtual and real-life interpersonal relationships. Meanwhile, recent studies have found that 29% of university students have SNS addiction and that SNS addiction is associated with anxiety, depression, and mania symptoms, similar to prior studies that investigated excessive SNS use [25,26].

Although recent studies have identified associations between excessive SNS use, SNS addiction, and mental health status, it remains unknown whether there is a clear pathway from excessive SNS use to SNS addiction, thus influencing users’ mental health (ie, pathway). As SNS use patterns can be differentiated by their social contexts (eg, different cultural values in interpersonal relationships on SNSs [27]), excessive SNS use and SNS addiction can be influenced by the users’ psychosocial status (ie, the influence of social factors on one’s mind [28]), such as the influence of social isolation and loneliness precipitating SNS use [29-31], while conversely influencing their mental health. Moreover, the possible mediating effect of SNS addiction on the pathway has been deduced as SNS addiction is also associated with SNS use patterns, psychosocial status, and mental health status [32-34]. Therefore, this study aimed to develop a structural equation modeling (SEM) approach to testing a hypothetical model and the potential mediating role of SNS addiction among Chinese university students in HK.

**Figure 1:**
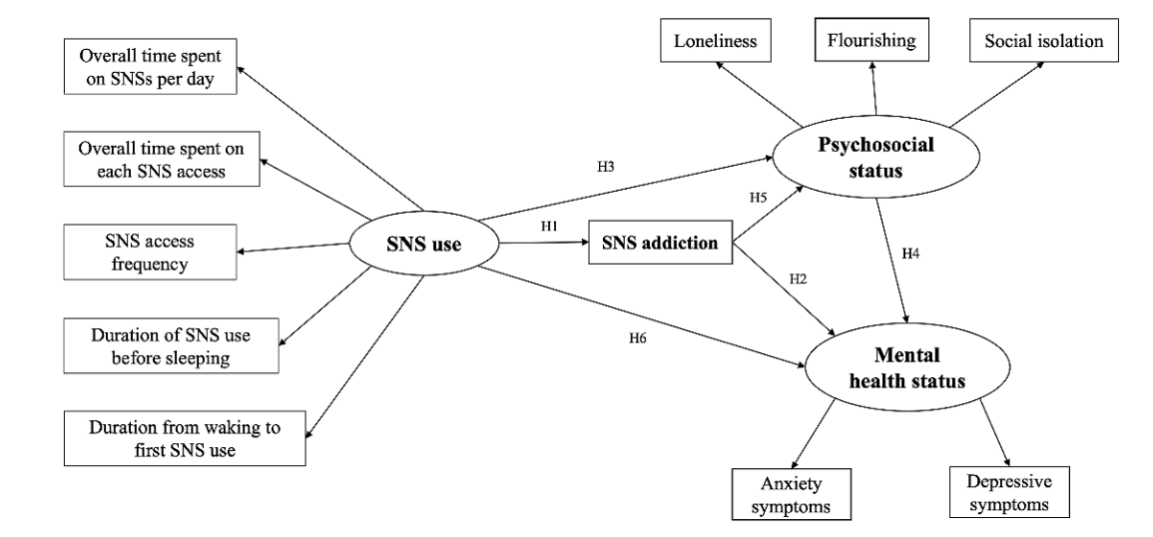
Conceptual model and hypotheses. Ovals represent unobserved latent variables. Rectangles represent observed measured variables. SNS: social networking service.

Based on the existing literature and knowledge gap, we developed the following hypotheses for this study and a hypothetical model (Figure 1):

- Hypothesis 1 (H1): SNS use is positively associated with SNS addiction.
- Hypothesis 2 (H2): SNS addiction is negatively associated with mental health status.
- Hypothesis 3 (H3): SNS use is negatively associated with psychosocial status.
- Hypothesis 4 (H4): Psychological factors are positively associated with mental health status.
- Hypothesis 5 (H5): SNS addiction is negatively associated with psychosocial status.
- Hypothesis 6 (H6): SNS use is negatively associated with mental health status.

## Methods

### Study Design and Sampling

A cross-sectional study design was adopted. An online survey link (Google Form) and a QR code printed on survey invitation flyers were distributed in busy public areas (eg, the entrance of library and cafeteria) within 2 major public university campuses in HK from February to March 2019. The online survey website included the study aim, respondents’ rights, and participation incentive (50 respondents were randomly selected through a lucky draw for a HK $25 [around US $3.2] cash voucher). The respondents were also invited to freely share the survey link with their peers from any HK university (ie, convenience and snowball sampling). The inclusion criteria were enrolled university students residing in HK with at least 1 SNS account. Exchange students from outside HK and respondents who did not answer more than 10 survey questions were excluded. To prevent multiple responses and confirm their identity as university students, each respondent’s mobile phone number and university email address were collected.

### Measurements

#### Demographics and SNS Use

Demographic characteristics, including sex, age, university, degree pursued, and academic performance, were investigated. The respondents’ patterns of SNS use (eg, overall time spent on SNS per day, time spent on each SNS access, SNS access frequency, and SNS use duration before sleeping and after waking) were surveyed.

#### SNS Addiction

SNS addiction was measured using a revised version of the Bergen Facebook Addiction Scale (BFAS), which consists of 6 questions [35,36]. The BFAS presented 0.83 internal consistency and reported convergent validity [35]. The overall score of the BFAS ranged from 6 to 30, with higher scores indicating a higher level of SNS addiction. The term “Facebook” in each item was changed to “SNSs” to include a wider range of SNSs, similar to Andreassen et al [36]. The internal consistency (Cronbach α) of the BFAS in this study was also 0.83.

#### Mental Health Status

The Patient Health Questionnaire-4 (PHQ-4) was used to screen for psychological symptoms among the respondents. The PHQ-4 consists of 2 domains (ie, depression and anxiety), and each domain has 2 questions [37]. The total score in each domain ranges from 0 to 6. Higher scores in the 2 domains refer to higher levels of depressive and anxiety symptoms, respectively. The internal consistency of the 2 domains was 0.82 and 0.81, respectively, in the original study [37] and 0.79 and 0.86, respectively, in this study.

#### Psychosocial Status

The respondents’ psychosocial status was measured according to their psychological well-being, social isolation, and loneliness. First, the Flourishing Scale was adopted to measure the respondents’ subjective psychological well-being [38]. The scale consists of 8 questions with an overall score range from 8 to 56, and higher scores represent higher subjective psychological well-being. The Flourishing Scale showed an internal consistency as 0.87 and convergent validity [38]. The internal consistency in this study was 0.92. Second, we adopted the Lubben Social Network Scale (LSNS-6) consisting of 6 questions to investigate the respondents’ social isolation level [39]. The overall score ranges from 0 to 30, and higher scores indicate lower levels of social isolation [39]. The internal consistency of the LSNS-6 was 0.83 in the original study and 0.75 in this study. Lastly, a brief loneliness scale [40] was also adopted to measure the respondents’ feelings of loneliness. The scale consists of 3 questions, and the total score ranges from 3 to 9. The internal consistency of the loneliness scale in the original research [40] and this study was 0.72 and 0.89, respectively.

### Statistical Analysis

Descriptive analysis was conducted to detail the respondents’ information about demographic characteristics, SNS use and addiction, and psychological profile. For continuous variables, the mean and SD were used, and for categorical variables, the frequency and percentage were used for analysis. Multiple linear regression was performed to assess the association between SNS use and SNS addiction. Structural equation modeling (SEM) was performed to examine the pathways from SNS use to mental health status. Multiple imputation was performed for missing data. Total, direct, and indirect effects in the hypothesized model were estimated using the maximum likelihood and bias-corrected (BC) 95% CI by the bootstrapping method with 2000 replications. Fitness indices of the SEM were considered, including the root-mean-square error of approximation (RMSEA; suggested close to or smaller than 0.06), the comparative fit index (CFI; suggested close to or larger than 0.95), the Tucker-Lewis index (TLI; suggested close to or larger than 0.95), and the standardized root-mean-square residual (SRMR; suggested close to or smaller than 0.08) [41,42]. IBM SPSS 24.0 and Amos 26.0 were used for all analyses at a .05 significance level.

## Results

### Respondents’ Characteristics

Of the 533 respondents, 483 (90.6%) who met the eligible criteria were included in this study. The mean age was 20.87 years (SD 2.68), and 66.9% (n=323) of the respondents were female. The majority (459/483, 95.0%) were from University Grants Committee (UGC)-funded universities (ie, large public universities) and undertook higher diploma or undergraduate courses (466/483, 96.5%). With regard to SNS use, 33.7% (163/483), 42.2% (225/483), and 31.0% (165/483) of the respondents used SNSs for over 1-2 h/day, 6-15 min each time, and every 31-60 min, respectively. In addition, 46.9% (250/483) and 36.0% (192/483) of them used SNSs for 31-60 min before sleeping and within 5 min or less after waking up. The mean scores for anxiety symptoms, depressive symptoms, flourishing, social isolation, and loneliness were 2.86 (SD 1.62), 2.70 (SD 1.58), 38.87 (SD 7.42), 16.90 (SD 5.06), and 5.55 (SD 1.79), respectively. The mean score for SNS addiction was 16.4 (SD 5.03); see Table 1.

**Table 1:** Demographic characteristics and frequency of SNS^a^ use and SNS addiction (N=483).

Variables		n (%)	SNS addiction, mean (SD)	SNS addiction, *P* value^b^
**Sex (*P*=.01)**				
	Male	160 (33.1)	15.49 (5.15)	—^c^
	Female	323 (66.9)	16.84 (4.92)	—
**University (*P*=.76)**				
	UGC^d^ funded	459 (95.0)	16.41 (0.06)	—
	Non-UGC funded	24 (5.0)	16.08 (4.63)	—
**Degree of study (*P*=.002)**				
	Higher diploma and undergraduate	466 (96.5)	16.53 (5.03)	—
	Postgraduate	17 (3.5)	12.65 (5.35)	—
**Academic performance (*P*<.001)**				
	First-class honors/quartile 4 or equivalent	84 (17.1)	14.92 (5.03)	—
	Second-class honors/quartile 3 or equivalent	192 (39.8)	15.89 (4.90)	—
	Second-class honors/quartile 2 or equivalent	118 (24.4)	18.03 (4.53)	—
	Third-class honors/quartile 1 or equivalent	20 (4.1)	17.55 (0.90)	—
	Missing	69 (14.3)	—	—
**Overall time spent on SNS per day (*P*<.001)**				
	≤1 h	72 (13.5)	12.43 (4.84)	—
	>1-2 h	163 (33.7)	16.42 (4.60)	—
	>2-3 h	125 (25.9)	18.40 (4.67)	—
	>3 h	123 (25.5)	20.29 (4.85)	—
**Time spent on each SNS access (*P*<.001)**				
	5 min or less	122 (22.9)	14.89 (5.05)	—
	6-15 min	225 (42.2)	16.10 (4.84)	—
	16-30 min	100 (18.8)	17.56 (4.60)	—
	31 min or more	36 (6.7)	20.08 (4.94)	—
**SNS access frequency (*P*<.001)**				
	Every 5 min or sooner	30 (5.6)	17.73 (6.73)	—
	Every 6-30 min	141 (26.5)	17.85 (4.32)	—
	Every 31-60 min	165 (31.0)	16.42 (4.77)	—
	Every 61 mins or later	147 (27.7)	14.69 (5.08)	—
**Duration of SNS use before sleeping (*P*<.001)**				
	61 min or more	43 (8.1)	14.03 (5.02)	—
	31-60 min	94 (17.6)	16.28 (4.55)	—
	6-30 min	250 (46.9)	18.49 (4.28)	—
	5 mins or less	74 (13.9)	19.26 (5.32)	—
**Duration from waking up to first SNS use (*P*=.02)**				
	5 min or less	192 (36.0)	16.83 (5.12)	—
	6-30 min	178 (33.4)	16.92 (4.72)	—
	31-60 min	64 (12.0)	15.77 (4.57)	—
	61 min or more	34 (6.4)	14.29 (5.45)	—
**Psychosocial status and mental health status**				
	Loneliness	—	5.55 (1.79)	<.001
	Flourishing	—	38.87 (7.42)	<.001
	Social isolation	—	16.90 (5.06)	<.001
	Anxiety symptoms	—	2.86 (1.62)	<.001
	Depressive symptoms	—	2.70 (1.58)	<.001

^a^SNS: social networking service.

^b^*P* value for the *t* test (1 or 2 groups) or ANOVA (3 groups or more).

^c^Not applicable.

^d^UGC: University Grants Committee. UGC-funded universities in HK including the University of Hong Kong (350/459, 76.3%), the Chinese University of Hong Kong (14/459, 3.1%), the Hong Kong University of Science and Technology (3/459, 0.6%), the Hong Kong Polytechnic University (81/459, 17.6%), the Education University of Hong Kong (0), the City University of Hong Kong (7/459, 1.5%), the Hong Kong Baptist University (3/459, 0.6%), and Lingnan University (1/459, 0.2%).

### Associations of SNS Addiction With SNS Use

SNS addiction was significantly associated with SNS use patterns (Table 2). A longer amount of time on SNSs per day (ie, >3 h), a longer amount of time on SNSs each access (ie, ≥31 min), and a higher frequency of SNS access (ie, less than every 30 min) were associated with a higher level of SNS addiction (adjusted beta [aβ]=6.03, 95% CI 4.66-7.40; aβ=4.99, 95% CI 3.14-6.83; and aβ=5.89, 95% CI 4.14-7.64, respectively). A longer duration of SNS use before sleeping (ie, ≥61 min) and a shorter duration from waking to first SNS use (ie, ≤5 min) were significantly associated with a higher level of SNS addiction (aβ=5.92, 95% CI 4.19-7.65 and aβ=3.27, 95% CI 1.73-4.82, respectively).

**Table 2:** Linear regression for the association between SNS^a^ use and SNS addiction.

Model		Crude			Adjusted^b^	
		β (95% CI)	β	β (95% CI)		β
**Overall time spent on SNSs per day**						
	≤1 h	REF^c^	—^d^	REF		—
	>1-2 h	3.35 (2.07-4.64)^e^	0.32	3.24 (1.94-4.54)^e^		0.30
	>2-3 h	4.82 (3.47-6.17)^e^	0.42	4.63 (3.26-6.00)^e^		0.40
	>3 h	6.23 (4.88-7.58)^e^	0.54	6.03 (4.66-7.40)^e^		0.52
**Time spent on each SNS access**						
	≤5 mins	REF	—	REF		—
	6-15 mins	1.21 (0.14-2.28)^f^	0.12	1.08 (–0.00 to 2.16)		0.11
	16-30 mins	2.67 (1.38-3.95)^e^	0.21	2.42 (1.07-3.76)^e^		0.19
	≥31 mins	5.19 (3.38-7.00)^e^	0.27	4.99 (3.14-6.83)^e^		0.26
**SNS access frequency**						
	≥Every 6 h	REF	REF	REF		REF
	Every 3 h	3.34 (1.51-5.17)^e^	0.28	3.29 (1.48-5.11)^e^		0.28
	Every 1 h	4.28 (2.52-6.04)^e^	0.40	4.21 (2.47-5.96)^e^		0.40
	≤Every 30 min	5.72 (3.96-7.48)^e^	0.54	5.89 (4.14-7.64)^e^		0.56
**Duration of SNS use before sleeping**						
	≤5 min	REF	—	REF		—
	6-30 min	2.92 (1.81-4.02)^e^	0.29	2.83 (1.69-3.98)^e^		0.28
	31-60 min	5.12 (3.79-6.46)^e^	0.40	5.10 (3.70-6.51)^e^		0.40
	≥61 min	5.89 (4.20-7.58)^e^	0.33	5.92 (4.19-7.65)^e^		0.34
**Duration from waking to first SNS use**						
	≥61 min	REF		REF		
	31-60 min	2.17 (0.33-4.02)^f^	0.15	2.15 (0.32-3.99)^f^		0.15
	6-30 min	3.33 (1.76-4.90)^e^	0.32	3.28 (1.73-4.84)^e^		0.31
	≤5 min	3.24 (1.69-4.80)^e^	0.32	3.27 (1.73-4.82)^e^		0.32

^a^SNS: social networking service.

^b^Adjusted for sex, age, academic performance.

^c^REF: reference group.

^d^Not applicable.

^e^*P*<.001.

^f^*P*<.05

### Mediating Effects of SNS Addiction Between SNS Use and Mental Health Status

**Figure 2:**
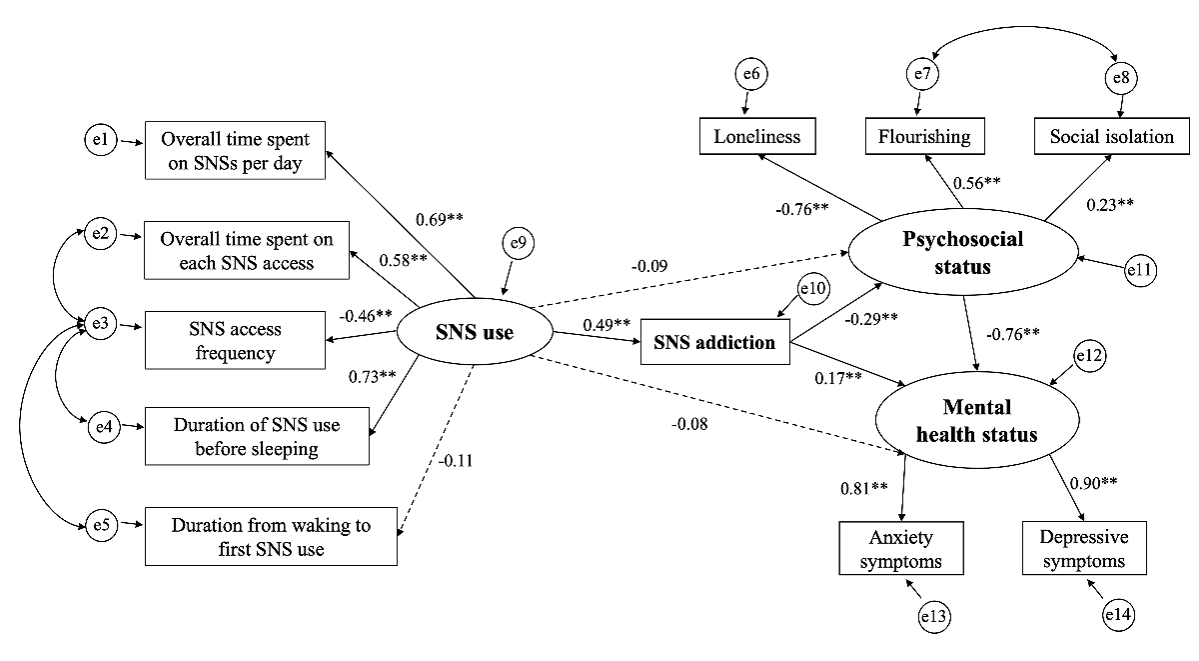
Standardized regression coefficient (β) of all variables in the hypothesized model. Ovals represent unobserved latent variables. Rectangles represent observed measured variables. Values are standardized path coefficients. **P<.01. SNS: social networking service.

The hypothesized model showed good fitness indices, with X^2^/*df*=2.16, RMSEA=0.05, CFI=0.97, TLI=0.95, and SRMR=0.04. Results from bootstrapping showed that SNS addiction has a significant mediating effect on the relationship between SNS use and psychosocial status, and mental health status (Figure 2).

H1 (SNS use and SNS addiction; β=0.49, 95% CI 0.39-0.58), H2 (SNS addiction and mental health status; β=0.17, 95% CI 0.06-0.27), H4 (psychosocial status and mental health status; β=–0.76, 95% CI –0.88 to –0.64), and H5 (SNS addiction and psychosocial status; β=–0.29, 95% CI –0.42 to –0.16) were supported. In contrast, H3 (SNS use and psychosocial status; β=–0.09, 95% CI –0.24 to 0.06) and H6 (SNS use and mental health status; β=–0.08, 95% CI –0.21 to 0.03) were not supported (Table 3).

**Table 3:** Standardized coefficients of raised hypotheses in the hypothesized model.

Hypothesis	β	BC^a^ 95% CI	*P* value
H1: SNS^b^ use → SNS addiction	0.49	0.39-0.58	.001
H2: SNS addiction → mental health status	0.17	0.06-0.27	.004
H3: SNS use → psychosocial status	–0.09	–0.24 to 0.06	.21
H4: Psychosocial status → mental health status	–0.76	–0.88 to –0.64	.002
H5: SNS addiction → psychosocial status	–0.29	–0.42 to –0.16	.001
H6: SNS use → mental health status	–0.08	–0.21 to 0.03	.16

^a^BC: bias-corrected.

^b^SNS: social networking service.

Three indirect pathways between SNS use and mental health status in SEM were investigated (Tables 4 and 5). The indirect effect of SNS addiction was significant on the pathway of SNS use to mental health status (β=0.20, 95% CI 0.08-0.34), but the indirect effect of psychosocial status was insignificant on the pathway (β=0.17, 95% CI –0.10 to 0.47). The indirect effect mediated by SNS addiction and psychosocial status in the pathway also showed a significant impact (β=0.26, 95% CI 0.14-0.44), and this pathway showed a stronger correlation than the pathway with SNS addiction alone as the mediator. The total indirect effect between SNS use and mental health status was significant (β=0.63, 95% CI 0.37-0.93). The direct effect of SNS addiction and psychosocial status on mental health status was insignificant (β=–0.19, 95% CI –0.49 to 0.08). Furthermore, the total effect of SNS addiction and psychosocial status that mediated the path from SNS use to mental health status was significant (β=0.44, 95% CI 0.19-0.72).

**Table 4:** Bootstrapping analyses to examine the indirect effect estimates of pathways in the hypothesized model.

Indirect effect	β	Product of coefficients			BC^a^ 95% CI		*P* value
		SE	Z				
SNS^b^ use → SNS addiction → mental health status	0.20	0.07	3.02	0.08-0.34		.003	
SNS use → psychosocial status → mental health status	0.17	0.14	1.20	–0.10 to 0.47		.21	
SNS use → SNS addiction → psychosocial status → mental health status	0.26	0.08	3.40	0.14-0.44		.001	

^a^BC: bias-corrected.

^b^SNS: social networking service.

**Table 5:** Total, direct, and indirect effects of mediation analysis in the model.

Effect	β	Product of coefficients		BC^a^ 95% CI	*P* value
		SE	Z		
Total	0.44	0.14	3.22	0.19-0.72	.002
Direct	–0.19	0.15	1.34	–0.49 to 0.08	.17
Indirect	0.63	0.14	4.52	0.37-0.93	.001

^a^BC: bias-corrected.

## Discussion

### Principal Findings

This study found that longer and more frequent use of SNSs is significantly associated with SNS addiction. This study also identified that SNS addiction and psychosocial status significantly and positively mediate the relationship between SNS use and mental health status (anxiety and depressive symptoms) in Chinese university students in HK.

#### SNS Addiction–Related Factors

SNS addiction significantly differed across the respondents’ demographics in this study. We found that females have a higher level of SNS addiction than males, corresponding with another study from HK [43]. In contrast, several studies from Europe have reported that males have a higher SNS (Twitter) addiction level and SNS obsession than females [44-46]. Although there are limited studies investigating the sex differences in SNS addiction, the distinct findings in the HK studies may be due to sociocultural influences on SNS use and addiction. Chinese females who are influenced by a collectivism-dominant culture tend to be introverted and gravitate toward indirect communication styles [47]. Accordingly, they prefer to use SNSs as a way to make emotional connections and maintain their existing interpersonal relationships [48]. They also tend to spend more time on SNSs than males [47,48]; thus they are more exposed to the risk of SNS addiction. An in-depth qualitative study and meta-analysis would be helpful to uncover the extent of sociocultural influences on SNS addiction.

We also found that SNS addiction is significantly higher among undergraduate students and those with poor academic performance, consistent with previous findings on academic performance [49,50] and undergraduate studies [51]. As time spent on SNSs for entertainment is a negative predictor of time spent studying [52] and academic performance, such as grade point averages [51], excessive and addictive SNS use for entertainment would decrease the time on education, resulting in low academic performance [44]. Although the associations of SNS use patterns with SNS addiction are little known, the associations between problematic uses of smartphones or the internet, and addictive behaviors have been identified in the literature. Excessive use or habitual checking of the smartphone can lead to smartphone addiction [53-55]. Similarly, long durations of time spent on the internet are a significant indicator of internet addiction [56]. Moreover, previous studies have reported that young adults with internet and smartphone addiction mainly spend time on the use of SNS websites and applications [57,58]. Given that SNSs can be used over the internet through smartphones and other electronic devices, and that internet and smartphone addiction results in similar user behaviors, our findings on SNS addiction can be comparable to studies investigating internet use on smartphones or internet addiction. Moreover, shorter durations from waking to first SNS use and longer durations before sleeping are strong indicators of SNS addiction [53,59]. These findings from previous studies resembled our findings regarding the association of excessive SNS use patterns with SNS addiction. We also further found the effect of the SNS use patterns on SNS addiction, namely that excessive SNS use can cause SNS addiction.

Although excessive SNS use can cause SNS addiction behaviors, young people are poor at recognizing their own SNS use as excessive. In 1 study that surveyed the effects of Facebook use on the social life and behaviors of 1000 university students in Pakistan, nearly 70% of those who showed addictive Facebook use did not discern themselves as having Facebook addiction [60]. Similarly, another study that collected SNS information on 804 adolescents in India revealed that only 4% perceived their SNS use to be highly problematic [61]. Opportunities exist for health professionals to guide young people to recognize their own excessive SNS use that can cause SNS addictive behaviors. Practical screening for SNS addiction can be assessed as a daily routine in the community when dealing with young people who show problematic and excessive SNS use. This may be the first step in recognizing potential cases before they develop further into SNS addiction. Concerning previous treatment for internet addiction [62], interventions conducted through a cognitive behavioral approach may also be helpful for users with SNS addiction in order to reconstruct their thoughts and behaviors and mitigate mental distress stemming from SNS addiction.

#### Impact of SNS Addiction on Mental Health Status

We found that SNS addition significantly influences mental health status (ie, anxiety and depressive symptoms), while the effect of SNS use patterns on mental health status is not significant. It should be noted that the difference between SNS use and SNS addiction is that the latter reflects more problematic behaviors and can lead to more severe outcomes that are associated with SNSs [7]. General SNS use may not be a potential factor; instead, it is the excessive use of SNSs that influences addictive behavior and mental distress [63]. Similar to our results, previous studies have reported that SNS addiction is associated with negative mental health outcomes, including depression, anxiety, and affective disorder [26,64,65]. However, these studies have mainly focused on the bivariate association between SNS addiction and mental health, while the effect of SNS addiction on mental health remains uncertain; that is, whether mental problems lead to SNS addiction or vice versa.

Furthermore, we identified that SNS addiction plays a mediating role in the pathway from SNS use to mental health status. This finding resonates with 1 study that identified that Instagram (an SNS) use predicts depression [25]. However, the mediating role of SNS addiction was not significant in that study, in contrast with our findings. SNS use in that study was only measured by 1 item (ie, the time spent on Instagram) [25], while we included diverse SNS use patterns (ie, 4 durations and 1 frequency measurement of SNS use). We also included all types of SNS use, but the aforementioned study only included Instagram use, although the use of different SNSs would be differently associated with SNS addiction and depression [25]. Therefore, the findings from our study offer more holistic and integrative viewpoints on the pathway from SNS use to mental health status via SNS addiction.

Of note, SNS use in moderation can positively affect users’ mental health status. SNS use can reinforce a user’s online relationships and solidify their offline connections, and this may, in turn, reduce their negative feelings and emotions (eg, anxiety, depression), positively influencing their mental health status [66,67]. Unfortunately, when SNS use becomes excessive, it conversely has an adverse effect on users. Although excessive SNS use is a popular concept in SNS studies and is known as a predictor of SNS addiction [68,69], there is ambiguity in the cut-off between non-excessive and excessive use, making excessive SNS use difficult to define. Therefore, SNS addiction would be more suitable than excessive SNS use to objectively determine that a user’s SNS use is problematic and to understand its influence on health outcomes [61,69]. Moreover, the sense of belongingness (feeling of being accepted in groups) is high among young people, particularly from East Asian countries that have collectivism-dominant societies. The sense of belongingness is positively associated with social and psychological functioning [70,71]. The characteristics of young people and the social environment in HK (ie, a high sense of belongingness that we measured in this study using social well-being, loneliness, and isolation scales) likely mediate SNS use, SNS addiction, and mental health status.

As an indispensable element of today’s leisure culture, absolute abstinence from accessing SNSs is not an appropriate treatment for SNS addiction and mental distress. Restricting the excessive use of SNSs, addressing the importance of SNS addiction control to improve psychosocial and mental health status and preventing relapse by encouraging self-reflection on SNS use may be possible solutions for designing educational programs. Particularly, during the COVID-19 pandemic, which hinders face-to-face intervention delivery, internet-delivered interventions (eg, internet-delivered cognitive behavioral therapy) can be also considered to address SNS addiction. Future studies on determining the appropriate duration of SNS use with findings transferable to practice guidelines would be needed.

### Limitations

This study had some limitations. Cross-sectional data in this study could not provide the causality between SNS addiction and mental health status. Discussions of the magnitude of the relationship between 2 elements (SNS addiction and mental health status) were inconclusive, with different opinions that this relationship can be bidirectional [72,73]. Therefore, more studies with longitudinal designs are needed to explore the direction and mechanism of causality for the relationship between SNS addiction and mental health status in the future. Additionally, as most of our participants were ethnic Chinese in HK, replication of our study for young adults in other sociocultural settings is recommended to reveal a more comprehensive relationship that was discovered through this study. Lastly, the participants responded to an online survey. Self-reported data may result in recall bias.

### Conclusion

This study provided novel information about the patterns of SNS use and its association with SNS addiction among university students. Findings from SEM also addressed that there is a significant mediating effect of SNS addiction between SNS use and mental health status, including anxiety and depressive symptoms. Further studies are suggested to demonstrate causal relationships with longitudinal data. This study helps to provide preliminary solutions for reducing SNS addiction and mental problems by conducting interventions using cognitive-behavioral approaches.

## Abbreviations

aβ: adjusted beta
BC: bias-corrected
CFI: comparative fit index
DSM-5: Diagnostic and Statistical Manual 5th Edition
HK: Hong Kong
ICD-11: International Classification of Diseases 11th Revision
RMSEA: root-mean-square error of approximation
SEM: structural equation modeling
SNS: social networking service
SRMR: standardized root-mean-square residual
TLI: Tucker-Lewis index
UGC: University Grants Committee
